# Supplementary material for: Human ApoA-I Overexpression Enhances Macrophage-Specific Reverse Cholesterol Transport but Fails to Prevent Inherited Diabesity in Mice
Source: Int J Mol Sci. 2019 Feb 2;20(3):655. doi: 10.3390/ijms20030655 (PMC6387412; doi:10.3390/ijms20030655)
Supplement: Supplementary file 1 [file ijms-20-00655-s001.pdf]

Human ApoA-I Overexpression Enhances Macrophage-Specific Reverse Cholesterol Transport but Fails to Prevent Inherited Diabesity in Mice

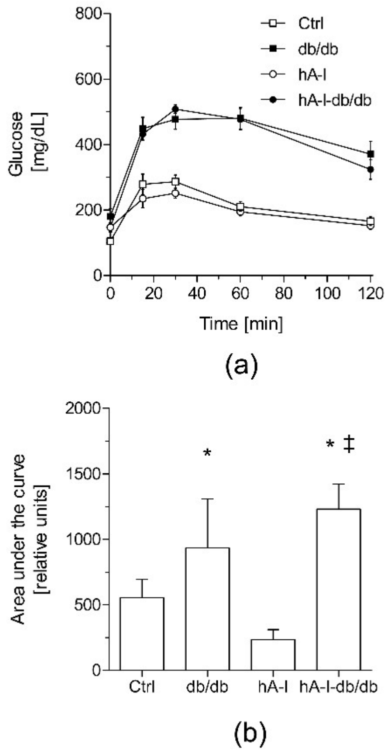

Supplementary Figure 1. Effects of hApoA-I overexpression on glucose tolerance. (a) Glucose kinetics following an intraperitoneal glucose tolerance test. (b) Area under the curve (AUC) of glucose after an intraperitoneal glucose injection. Values are presented as the means ± SEM from 5 individual animals per group.

SUPPLEMENTARY FIGURE 2

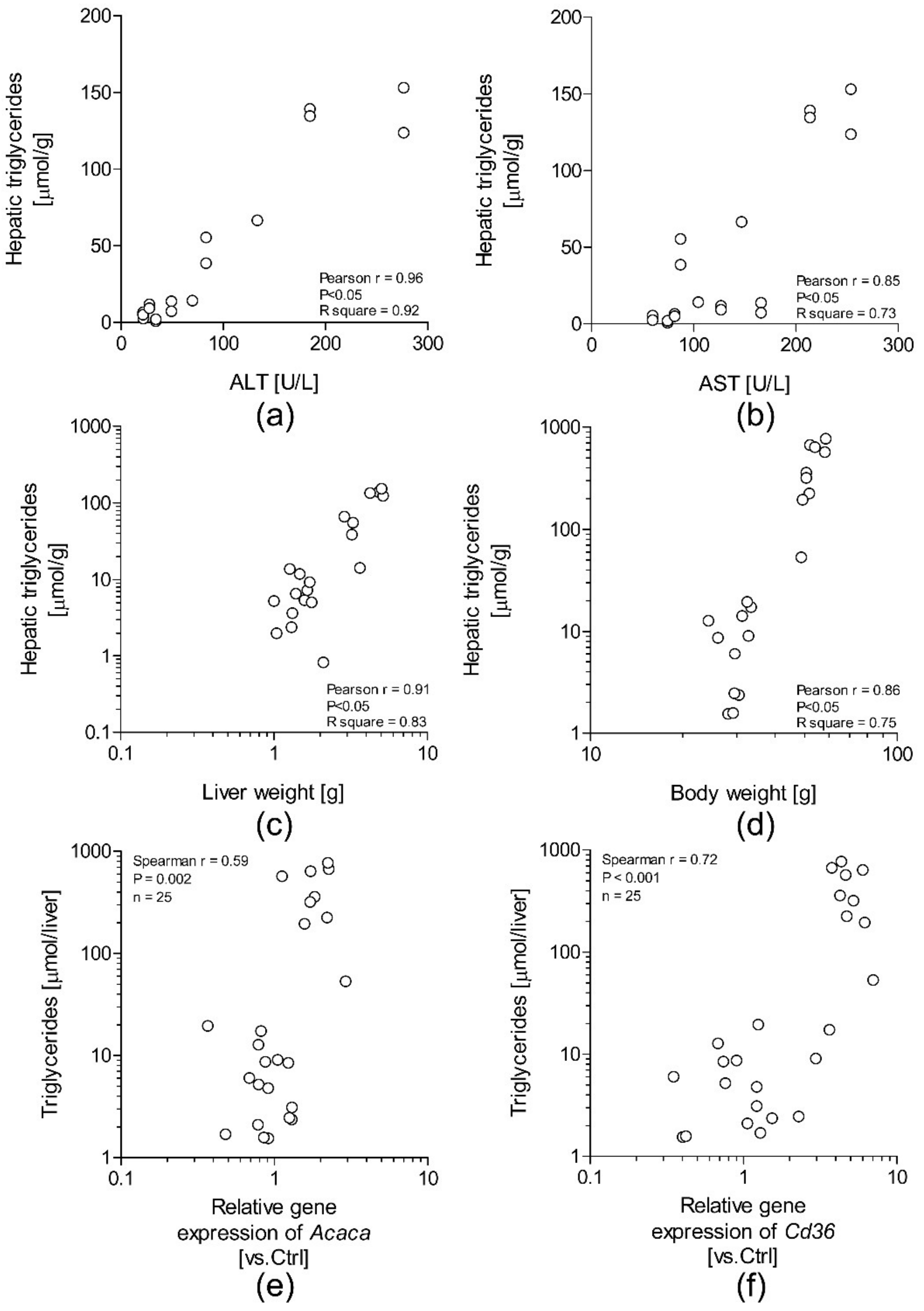

Supplementary Figure 2. Hepatic triglyceride contents and liver function. (a) Relationship between hepatic triglyceride levels and plasma ALT level. (b) Relationship between hepatic triglyceride contents and plasma AST levels. (c) Relationship between hepatic triglyceride contents and liver weight. (d) Relationship between hepatic triglyceride levels and body weight. (e) Relationship between hepatic triglyceride levels and *Acac* mRNA expression. (f) Relationship between hepatic triglyceride levels and *Cd36* mRNA expression. The data are reported as Spearman's r values. A nonparametric Spearman correlation test was used to study the extent of correlations between parameters.

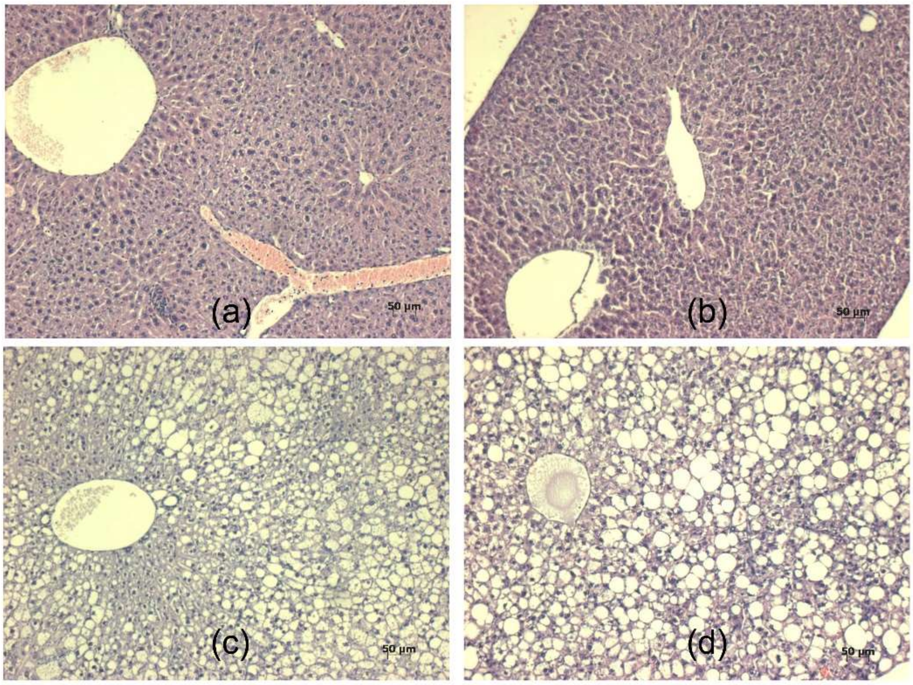

Supplementary Figure 3. Representative images of liver tissue sections stained with H&E. (a) Images of hematoxylin-eosin staining of liver tissues from Ctrl mice, (b) hA-I mice, (c) db/db mice, and (d) hA-I-db/db mice.. Scale bar: 50 µm. Note that livers from hA-I-db/db mice showed severe micro and macrovesicular steatosis compared with db/db mice.

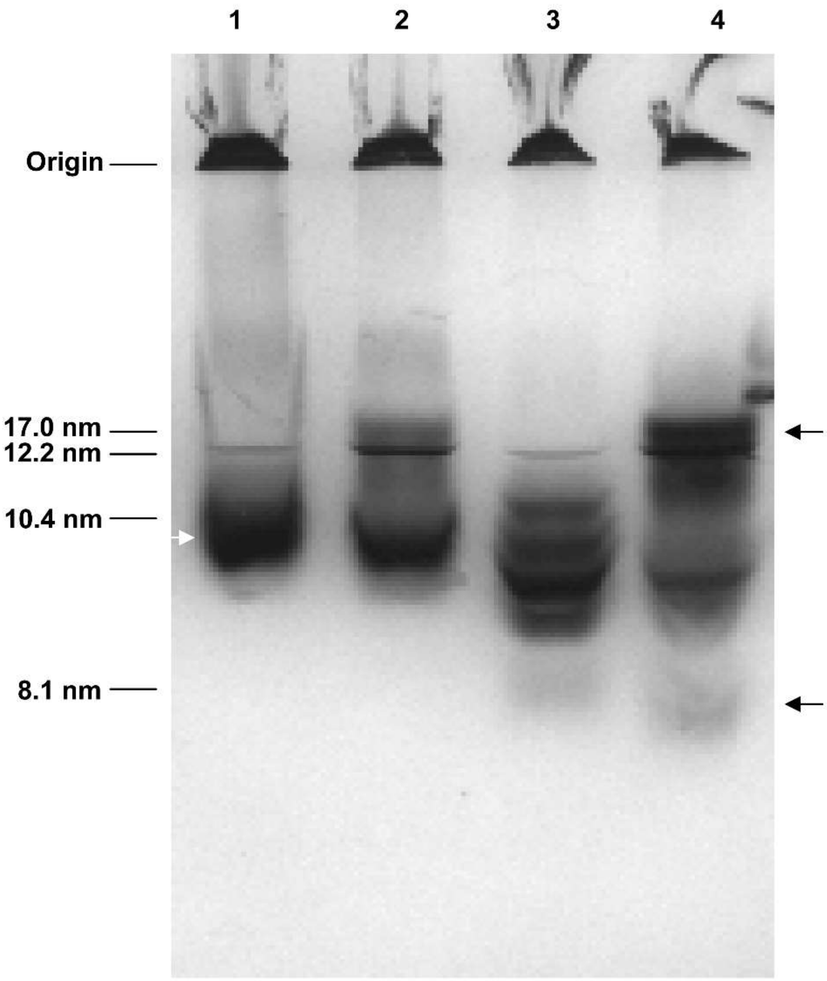

Supplementary Figure 4. Effect of hApoA-I overexpression on the size of plasma HDL. HDL was electrophoretically separated on nondenaturing 4-30% gradient polyacrylamide gels containing ApoB-depleted plasma samples that were prestained with Sudan black IV. The size of the molecular weight markers is shown on the left. A polydisperse pattern of HDL is shown in the hApoA-I-overexpressing mice. Lane 1, Ctrl mice; lane 2, db/db mice; lane 3, hA-I mice; lane 4, hA-I-db/db mice. The white arrow shows the monodisperse population of HDL from Ctrl mice. Black arrows indicate the appearance of larger and smaller HDL in hA-I-db/db mice compared with the complex HDL pattern present in hA-I mice (shown by the bracket).
